# A Divergent Variant of the Eleventh Human Polyomavirus Species, Saint Louis Polyomavirus

**DOI:** 10.1128/genomeA.00812-13

**Published:** 2013-10-24

**Authors:** Diana V. Pastrana, Peter C. FitzGerald, Giao Q. Phan, Manish T. Raiji, Philip M. Murphy, David H. McDermott, Daniel Velez, Valery Bliskovsky, Alison A. McBride, Christopher B. Buck

**Affiliations:** National Cancer Institute, Bethesda, Maryland, USAa; National Institute of Allergy and Infectious Diseases, Bethesda, Maryland, USAb

## Abstract

Saint Louis polyomavirus (STLPyV) was recently discovered in human feces. Using random-primed rolling circle amplification combined with deep sequencing, we have found a divergent variant of STLPyV in a sanitized human skin wart specimen. The result strongly suggests that STLPyV directly infects humans and is not simply a dietary contaminant.

## GENOME ANNOUNCEMENT

The number of known human polyomavirus (HPyV) species has been expanding rapidly in recent years. Several HPyVs, including BK polyomavirus (BKV or BKPyV), JC polyomavirus (JCV or JCPyV), Merkel cell polyomavirus (MCV or MCPyV), and trichodysplasia spinulosa-associated polyomavirus (TSV or TSPyV), are known to cause disease in immunocompromised individuals (1). Other recently discovered HPyV species have not yet been clearly associated with any disease.

Saint Louis polyomavirus (STLPyV) was recently discovered in human fecal samples collected in Malawi, the United States, and the Gambia (2). It is currently uncertain whether STLPyV is a bona fide human-tropic polyomavirus species or was instead derived from a dietary source. In this genome announcement, we report a divergent variant of STLPyV isolated from condylomas (skin warts) surgically removed from the buttocks of a patient suffering from the primary immunodeficiency warts hypogammaglobulinemia infections and myelokathexis syndrome (WHIMS). Observation of STLPyV in a surface-sanitized tissue specimen strongly suggests that STLPyV productively infects humans and thus can be considered the eleventh known HPyV.

We previously discovered HPyV10 in this same surgical sample (3). For HPyV10 identification, the warts were minced and treated with detergents and nucleases, and virions were purified out of the resulting extract by ultracentrifugation. DNA was extracted from the purified virions and subjected to random-primed rolling circle amplification (RCA) (Templiphi, GE). Restriction fragments of the RCA product were subjected to plasmid-based cloning. This cloning-based approach revealed the presence of three viral species: human papillomavirus type 6 (HPV6), HPV124, and HPyV10. For discovery of the STLPyV variant, a portion of the RCA product was processed using Nextera reagents (Illumina) and subjected to deep sequencing (Illumina, Miseq), generating a total of 269,313 paired-end-reads. Of these reads, ≃2% showed homology to known human or bacterial sequences when analyzed by BLASTn. Additionally, 198,606 HPV6-like reads, 22,745 HPV124-like reads, and 31,043 HPyV10-like reads were found. Sequences with close homology to HPV76 (1,505 reads), HPV123 (332 reads), and TSPyV (187 reads) were also observed. A total of 634 reads showed >90% homology to previously reported STLPyV isolates.

Based on the STLPyV-like reads, PCR primers were designed to perform inverse PCR using Herculase II Fusion DNA polymerase (Agilent). Two separate PCRs using different primer pairs each yielded an ~5-kb band. Each PCR product was purified and cloned into pCR-Blunt-II-TOPO (Invitrogen), and the captured inserts were sequenced by primer walking. Both clones showed identical sequences. Sequencing of a separate PCR confirmed the MiSeq reads used to design the inverse PCR primers. PASC analysis (http://www.ncbi.nlm.nih.gov/sutils/pasc/) showed that the complete genomic sequence of the new STLPyV-like isolate, which we named “11ww,” shares 92% homology with both the original MA138 (NC_020106) and WD972 (JX463184) isolates of STLPyV (2). STLPyV-11ww shows a pattern of open reading frames (ORFs) and splicing signals similar to MA138 (4). Based on current taxonomic conventions (5), this degree of homology qualifies isolate 11ww as the third known member of the viral species STLPyV.

### Nucleotide sequence accession number.

The complete genomic sequence of STLPyV-11ww was deposited in GenBank. It has been assigned the accession number KF525270.
